# The Entrapment of Somatostatin in a Lipid Formulation: Retarded Release and Free Radical Reactivity

**DOI:** 10.3390/molecules24173085

**Published:** 2019-08-25

**Authors:** Anna Vita Larocca, Gianluca Toniolo, Silvia Tortorella, Marios G. Krokidis, Georgia Menounou, Giuseppe Di Bella, Chryssostomos Chatgilialoglu, Carla Ferreri

**Affiliations:** 1 R&D Laboratory, Lipinutragen srl, Via Piero Gobetti 101, 40129 Bologna, Italy; 2 Institute of Nanoscience and Nanotechnology, N.C.S.R. “Demokritos”, 15310 Agia Paraskevi Attikis, Greece; 3 ISOF, Consiglio Nazionale delle Ricerche, Via P. Gobetti 101, 40129 Bologna, Italy; 4 Di Bella Foundation, Via G. Marconi 51, 40122 Bologna, Italy

**Keywords:** liposomal somatostatin, retarded delivery, free radicals, isomerization, trans lipid, peroxidation

## Abstract

The natural peptide somatostatin has hormonal and cytostatic effects exerted by the binding to specific receptors in various tissues. Therapeutic uses are strongly prevented by its very short biological half-life of 1–2 min due to enzymatic hydrolysis, therefore encapsulation methodologies are explored to overcome the need for continuous infusion regimes. Multilamellar liposomes made of natural phosphatidylcholine were used for the incorporation of a mixture of somatostatin and sorbitol dissolved in citrate buffer at pH = 5. Lyophilization and reconstitution of the suspension were carried out, showing the flexibility of this preparation. Full characterization of this suspension was obtained as particle size, encapsulation efficiency and retarded release properties in aqueous medium and human plasma. Liposomal somatostatin incubated at 37 °C in the presence of Fe(II) and (III) salts were used as a biomimetic model of drug-cell membrane interaction, evidencing the free radical processes of peroxidation and isomerization that transform the unsaturated fatty acid moieties of the lipid vesicles. This study offers new insights into a liposomal delivery system and highlights molecular reactivity of sulfur-containing drugs with its carrier or biological membranes for pharmacological applications.

## 1. Introduction

Somatostatin (also known as somatotropin release-inhibiting factor or growth hormone release-inhibiting factor, SST) is a cyclic peptide of 14 amino acids first isolated from ovine hypothalamic and known to inhibit the secretion of multiple hormones (e.g., growth hormone, insulin, glucagon, gastrin), gastric acid and pancreatic enzymes. In the central nervous system, this peptide acts as a neurotransmitter and affects locomotor activity and cognitive functions [1]. SST exerts its activity by binding to at least five different subtypes of specific receptors (SSTR 1-5) located on the target cells with a wide range of biological effects that can be exploited for the treatment of a variety of human diseases [2,3,4]. Signaling pathways activated by the SST-receptor interaction (such as, mitogen-activated protein kinase pathway, inhibition of adenylyl cyclase, activation of phosphotyrosine phosphatase, changes in plasma membrane calcium and potassium channel activity) are evoked for the antineoplastic and anti-proliferative activities [5,6]. The therapeutic potential of SST is strongly limited by its very short half-life of less than 1–2 min in plasma [7], as expected for several neuropeptide hormones that must be rapidly inactivated after their release and interaction with their receptors. In fact, in neuronal cell cultures the degradation of SST was measured, and membrane-bound proteases or proteases released into the incubation medium were found to be responsible for this inactivation [8]. 

**Figure 1:**
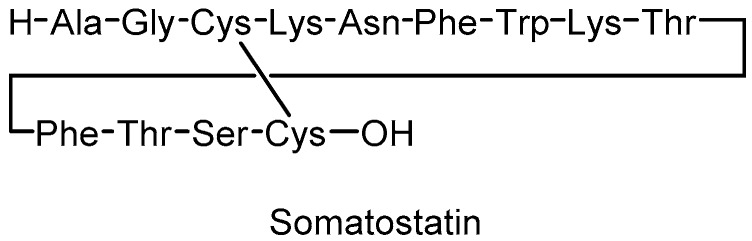
Amino acid sequence of the peptide somatostatin (SST).

Solutions to the short lifetime have been proposed based on two main approaches: (a) preparation of SST analogues attaching one or more groups to the peptide molecule, such as the N-methyl group, able to act as a shield for the in vivo hydrolysis [9]; (b) encapsulation of SST or its analogues in polymeric materials, such as poly(alkyl cyanoacrylate) nanocapsules [10], or in natural phospholipid vesicles, eventually coated with agents ensuring circulation in blood, such as polyethylene glycol (PEG), that protects liposomes from recognition and rapid removal from circulation by the phagocyte system [11]. SST, its analogue octreotide or other synthetic analogues are also studied in liposomal formulations combined with antitumoral drugs like daunorubicin or with radiopharmaceuticals, due to their ability to target SSTR-rich tumoral cells [12,13,14,15]. The biocompatibility and biodegradability of liposomes have an overwhelming importance thus motivating research to deepen the use of natural phospholipids as drug delivery components. It is worth noting that, as far as natural lipid formulations are concerned, the choice of the fatty acid quality is important for liposome behavior, since it influences fluidity and permeability properties connected with drug release [16]. In the frame of our interest in liposomes as tools for investigations in biomimetic chemistry, we recently proposed trans-double bond-containing liposomes for drug delivery systems with differentiated behavior respect to those made of natural unsaturated lipids, which display cis-geometry [17]. In fact, trans double bonds are well known to change the molecular properties of the lipid assembly, compared to the cis double bonds, shifting toward less fluid and permeable double lipid layers [18]. Moreover, the cis-trans double bond isomerization can occur as an endogenous process in cells, due to the formation of sulfur-centered radicals during oxidative stress and their reaction with unsaturated lipids [19]. Recently, lipid isomerization and peroxidation were observed in liposomes with iron-binding antitumoral drugs such as bleomycin in the presence of thiols [20]. We were intrigued by the SST structure, which contains the disulfide bond between Cys-3 and Cys14 (Figure 1) that under free radical conditions can produce thiyl radicals and react with unsaturated lipids. Such reactivity for SST has not yet been reported. 

Here we report the entrapment of SST in a liposome formulation with full characterization of size and properties including encapsulation efficiency, retarded release and the possibility of lyophilization-reconstitution of the emulsion. Liposomes containing SST and unsaturated fatty acid moieties were used also as biomimetic model of iron-induced free radical stress, evidencing the occurrence of lipid isomerization and peroxidation processes. This study contributes to new knowledge in the interdisciplinary field of liposomes and drug mechanism.

## 2. Results and Discussion

**Figure 2:**
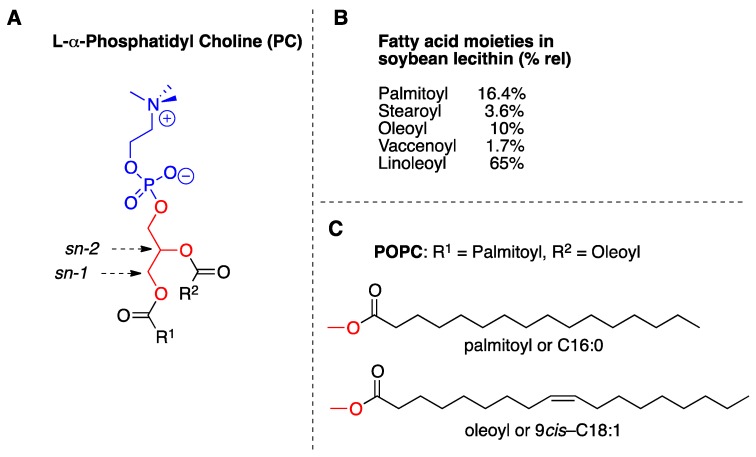
Lipid structures: (**A**) l-α-phosphatidyl choline (PC) general structure; (**B**) list of fatty acids present in soybean lecithin and the relative percentages obtained from the gas chromatographic analysis; (**C**) the structures of palmitic acid and oleic acid present in 1-palmitoyl-2-oleoyl phosphatidyl choline (POPC).

Lipid formulations used in this study were made of natural l-α-phosphatidyl choline (PC) from soybean lecithin, of general formula shown in Figure 2A, which has choline as hydrophilic head whereas the hydrophobic tails are composed by different fatty acids. In Figure 2B the fatty acid moieties of soybean lecithin are shown as relative percentages (%rel). In order to study the chemical behavior of SST, we used also the synthetic phospholipid 1-palmitoyl-2-oleoyl phosphatidyl choline (POPC), which contains the saturated fatty acid (SFA) palmitic acid (C16:0) and the monounsaturated fatty acid (MUFA) oleic acid (9*cis*-C18:1) (Figure 2C). 

Experiments of SST release and resistance to degradation were carried out with the soybean lecithin liposome preparations, exploring the behavior of the liposomes after lyophilization-reconstitution. POPC liposomes and soybean lecithin liposomes containing SST were then used as biomimetic models of metal-induced free radical stress to highlight the reactivity between peptide and unsaturated lipids.

Soybean lecithin liposomes were prepared for the release experiments of this study. l-α-phosphatidylcholine from soybean was dissolved in ethanol to reach 5 mM concentration, and this ethanolic solution was evaporated under vacuum in order to obtain a lipid film without traces of solvent. In tridistilled water, 0.1 M citrate buffer at pH 5 was prepared and added with 2 mM sorbitol. The peptide SST (0.5 mg/mL) was dissolved in this buffer. At this pH, closer to its isolectric point (pH = 5.6) [21], the peptide was shown to be stable as established by LC analyses at different time points (for methods see Experimental part). The peptide solution was added to the lipid film, calculating a lipid:peptide ratio of 20:1 in all preparations. After 5 min at the vortex, the suspension was characterized by DLS (Dynamic Light Scattering) for evaluating particle size and polydispersity index (PDI) prior to be lyophilized. The mean diameter was found to be 159.5 ± 12 nm (PDI 0.16 ± 0.01). The measurements of the nanoemulsion (NE) mean diameter were run in an interval time of 5 h, showing that the size does not undergo variations. It is worth noting that after lyophilization and reconstitution the mean diameter was found to be similar to the original preparation. The encapsulation efficiency (EE) in soybean lecithin liposomes was evaluated, resulting to be 66.9 ± 2.9%. Using liposomes formed by soybean lecithin two in vitro experiments were designed for the release of SST: in aqueous citrate buffer (pH 5) and, in order to test the resistance to peptidases, in human plasma.

### 2.1. Release Experiments in Aqueous Citrate Buffer 

For each set of experiments 5 mg of SST were used, and after lyophilization the suspension was reconstituted using 10 mL of tridistilled water. 

The release experiment was designed to analyze not only the directly released SST (in the aqueous phase of the nanoemulsion (NE)—direct analysis) but also the amount of non-released SST, still in the lipid droplets (indirect analysis). Since SST can have stability problems, the determination of the peptide remained in the lipid phase was meant to collect important additional information for the characterization of the release profile of the NE. The direct analysis consisted of measuring the concentration of SST in the aqueous fraction of the emulsion after centrifugation, using LC/MS analysis following a published protocol [22]. In the indirect analysis the peptide was extracted from the lipid droplets, separated by centrifugation, and analyzed again by LC/MS. 

**Figure 3:**
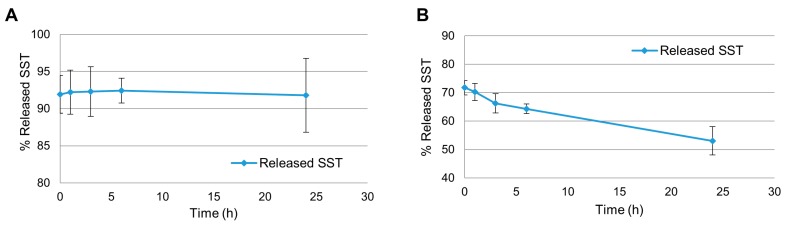
Released somatostatin (SST) from the nanoemulsions (NEs) following protocol 1 in 0.1 M buffer citrate pH 5.0. (**A**) % released SST considering the sum of the SST fraction found in aqueous phase (direct analysis) and the SST fraction in the lipid droplets (indirect analysis) at each time point. (**B**) % released SST in the aqueous phase considering as 100% the initial amount of SST used in the preparation procedure (0.5 mg/mL).

Moreover, the release experiments were run using the NE emulsions reconstituted from the lyophilized samples, following two different protocols. In Protocol 1 (data reported in Figure 3), the freeze-dried NEs containing SST (0.5 mg/mL) were reconstituted in water to recreate the same conditions of the original preparation and the samples were left for the indicated times; the quantities of SST at each time point were then calculated as shown in Figure 3A,B: (a) the first one from the analyses of each sample treated by centrifugation and then considering both SST quantities in the aqueous and lipid phases. In Figure 3A these two quantities give ca 93% of released SST which is constant along the time; (b) the second quantification considering the % SST released in the aqueous phase. In Figure 3B it is clearly shown that the peptide is diminishing along the time when considering as starting quantity the encapsulated SST (0.5 mg/mL). 

**Figure 4:**
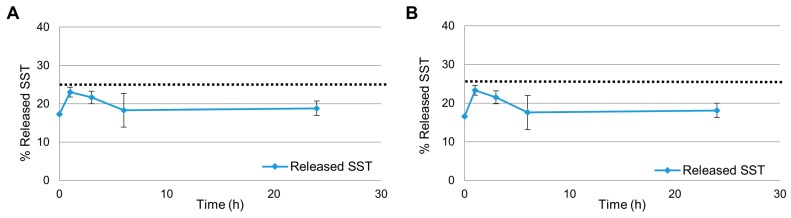
Released SST from the NEs following protocol 2. (**A**) Considering as 100% the sum of the fraction released, the fraction still encapsulated and the non-encapsulated one at each time point. (**B**) Considering as 100% the amount of SST expected in the samples from the centrifugation step. The dotted line represents 25%, the amount of SST available after the centrifugation to remove the non-encapsulated fraction of SST.

We also wanted to determine the SST released from the NEs with the removal of the aqueous phase replaced at each time point by fresh buffer. This led to the second protocol (Protocol 2—data in Figure 4) which involved the centrifugation step to remove the aqueous phase of the NE; the resulting SST-containing lipid pellet was then suspended in 0.1 M citrate buffer pH 5.0 and the release experiment was carried out at the indicated times. Again, in Figure 4A,B the SST quantities are reported in a complementary way: in Figure 4A it is calculated from the direct and indirect analyses, and in Figure 4B it is reported the release in buffer from the direct analysis. 

In all cases LC/MS using SST as calibration and standard reference as described in the Materials and Methods were carried out at the time points (0, 1 h, 3 h, 6 h, 24 h) when samples are collected and analyzed. The % of released SST were calculated considering as 100% the starting amount of SST used for the preparation of the NEs (0.5 mg/mL).

The two protocols gave clearer insight into the behavior of SST and release dynamics of the NE. In particular, the first protocol gave information on the behavior of the NE and the variations in the partition of SST between the two phases along the time; the second one informed on the diffusion of SST from the lipid droplets to the less concentrated aqueous phase. Also, since the experiments were run for 24 h, the stabilizing effect of NE on SST could be evaluated. 

In Figure 3A the SST quantity was found ca. 92% at time 0 and its concentration remained steady up to 24 h. Figure 3B shows that the SST released in the aqueous phase at time 0 was ca. 72% and decreased to 53% after 24 h. Interestingly, at time 0 the starting amount of SST was different in the two graphs: part of the SST was already unavailable at time 0, corresponding to roughly 22%, as it can be calculated considering a) the value at time 0 of ca. 92% given by the sum of the fraction released and the fraction in the lipid droplets, as shown in Figure 3A, and b) the value at time 0 of ca. 28% SST not found in aqueous phase from the initial SST concentration, as shown in Figure 3B. The results in Figure 3B also proved that some degradation/aggregation processes can occur to SST from its original preparation during lyophilization and manipulation steps, since there is a decrease in the peptide concentration found after reconstitution. Nevertheless, when SST was removed from the aqueous phase, part of the SST associated to the lipid phase was released and went to the aqueous phase, which is shown in Figure 3A, where the released/total ratio remained constant up to 24 h. By comparing the two graphs it is clear that, while some SST is degraded in the aqueous phase, some SST in the lipid droplets was released in order to maintain a constant presence of peptide in the aqueous fraction. Since SST is constantly released into the aqueous phase of the NE for 24 h, the system can be considered capable of releasing the peptide in a prolonged manner. 

In protocol 2 the freeze-dried NEs were first reconstituted using the aqueous phase and then centrifuged to remove the non-encapsulated fraction of SST, to understand the quantity of the drug immediately released by the droplets upon reconstitution. The pellet obtained after the centrifugation was suspended in buffer at pH 5 to start the release follow up. Again, the results of protocol 2 (Figure 4) were expressed in two ways. In Figure 4A 100% is represented by the sum of SST found with the direct analysis and the indirect analysis and in Figure 4B 100% was considered to be amount of SST used for the preparation of the NEs (0.5 mg/mL).

The aqueous phase removed by centrifugation contained 75% of the peptide in feeding, therefore only 25% was calculated for the release experiments (marked with the dotted line in Figure 4). There is no remarkable difference between Figure 4A,B: in both cases at initial time 17% of the total amount of SST was in the aqueous phase of the NE. After 1 h, the peak of concentration was reached in both conditions (23% of total SST, corresponding to 90% of the available amount). Afterward the concentration started to decrease, being 19% after 24 h (85% of the available drug). The similarity between Figure 4A,B suggests that none or very little degradation occurred during this experiment. Probably this is due to the overall lower concentration of the peptide, and consequently the stabilizing effect of the NE is more evident. Also, it is clear that upon reconstitution SST distributes between the two phases immediately and, apart from the peak at 1 h, the concentration of SST remains constant in both compartments for the whole experiment.

### 2.2. Release Experiments in Human Plasma

Only direct analysis was used to evaluate the concentration of SST in the experiments of the NE release using human plasma, since the indirect analysis proved to be unsuitable for the LC/MS system when working with plasma. Therefore, 100% of released SST corresponds to amount of SST used for the preparation of the NEs (0.5 mg/mL). 

**Figure 5:**
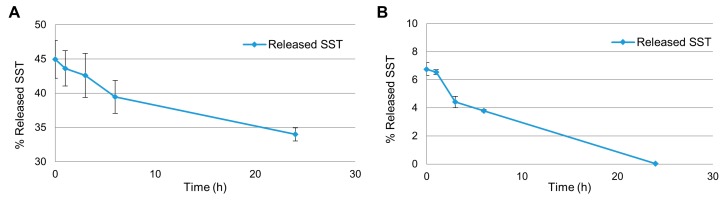
(**A**) Measurement of SST concentration in plasma after reconstituting the NEs directly in plasma (protocol 1). (**B**) Measurement of SST concentration in plasma after removing the non-encapsulated SST fraction (protocol 2). Percentages are calculated from the LC/MS analyses related to the starting concentration of 0.5 mg/mL.

The experiments were run following two protocols similar to the ones described above. The first procedure consisted of reconstituting the freeze-dried NE directly in plasma to start the release experiment (Figure 5A). In the second one, the NE was first reconstituted in water, then underwent centrifugation to remove the non-encapsulated fraction of SST, and the resulting pellet was suspended in human plasma to start the experiment (Figure 5B).

In Figure 5A, the amount of SST measured in the aqueous phase of the NE was 45% of the starting concentration (0.5 mg/mL) at time 0 and decreased to 34% after 24 h. When the NE release profile was studied following protocol 2, roughly 7% of the SST was found in plasma at time 0, and after 24 h only traces of SST were found (0.03%) (Figure 5B). These results imply that there was a decrease in concentration due to degradation of the peptide. Nevertheless, SST is known to have very short half-life in plasma [7] and the NE proved to be efficient in improving the peptide half-life, maintaining its measurable concentration at least for the first 7–8 h.

### 2.3. Reactivity of SST in Liposome Vesicles

We were interested in the study of the reactivity of the disulfide bridge present in the structure of SST, between the cysteine residues in positions 3 and 14 (Figure 1), since a variety of conditions can influence the reactivity of this functional group, such as the presence of redox-active metal ions. In fact, the chelation ability of metals toward disulfides is known, and it can act to generate catalytic amount of thiyl radicals together as known for Fe(III) and its reduction to Fe(II) [23]. Since we worked under aerobic conditions and it is known that complexes with Fe(II) can be oxidized to Fe(III) under air [24], we used Fe(II) and Fe(III) salts in our experiments in order to follow the liposome reactivity in both cases. On the other hand, as mentioned in the introduction, investigations by some of us demonstrated that iron binding in case of the antitumoral drug bleomycin was able to induce the formation of thiyl radicals, which react with liposome vesicles in terms of cis-trans isomerization and peroxidation of the unsaturated fatty acid moieties of vesicle lipids [20]. We also used the reduced form of SST (red-SST), with the free thiol groups in positions 3 and 14, which is commercially available, to compare the results of the two free cysteine residues with the cystine moiety of the natural peptide in the formation of iron-complex, including the role of thiyl radicals in the consequent isomerization reaction. As matter of facts, the reduction of SST is considered to occur in vivo and is associated to fibril formation relevant to the storage and secretion [25] but was never connected with chemical reactivity. 

**Figure 6:**
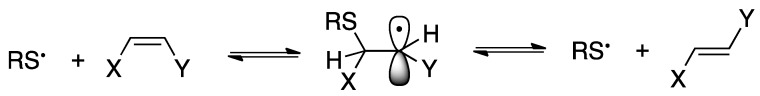
Reaction mechanism for the cis–trans isomerization of a double bond catalyzed by thiyl radicals.

We used the biomimetic model made of large unilamellar vesicle by the extrusion technique with 200 nm of diameter (LUVET) [26], in the presence of SST or red-SST for investigating membrane phospholipid behavior. Two different compositions of liposomes have been employed: first, the synthetic phospholipid, 1-palmitoyl-2-oleoyl phosphatidyl choline (POPC) was used to study the reactivity of the MUFA moiety of oleic acid (9*cis*-C18:1, Figure 2C) which is expected to be directly transformed by thiyl radical catalyzed cis-trans isomerization into its corresponding geometrical trans isomer, elaidic acid (9*trans*-C18:1) by the addition-elimination mechanism represented for the double bond in Figure 6. The second material used for liposome formation was soybean lecithin, containing various percentages of saturated, mono- and poly-unsaturated (MUFA and PUFA) fatty acids (Figure 2B). The PUFA residues of soybean lecithin liposomes can be transformed both by oxidative and isomerization pathways. In particular, linoleic acid (9*cis*,12*cis*-C18:2), the most representative PUFA in lecithin (65% of the total fatty acid composition), can give geometrical trans isomers that are separated, recognized and quantified by GC analysis [18,19,26,27]. Lipid peroxidation can be indirectly estimated by the amount of the PUFA linoleic acid in each experiment, evidencing its diminution by using the GC calibration curves vs. the saturated fatty residue of palmitic acid (C16:0), as previously described [20,28]. GC analysis is the gold standard for the fatty acid quantification. 

The iron salts used in the experiments were: ferrous (II) ammonium sulfate Fe(NH_4_)(SO_4_)_2_ and ferric (III) sulfate Fe_2_(SO_4_)_3_. The salts at concentration of 100 μM were added to the liposome suspension (2 mM) and then the addition of SST or red-SST (100 μM) to the vesicle aqueous suspension was carried out by syringe pump, keeping the vials at 37 °C in an orbital shaker for the indicated reaction time. The experiments were carried out in aerobic and anaerobic conditions and the results are summarized in Table 1. Blank experiments included liposome reaction without the peptide and in presence/absence of iron, in order to better estimate the drug contribution to lipid reactivity. The blank experiments without SST with POPC, and with or without Fe salts, gave a not detectable quantity of trans isomers (results not shown). 

With SST-Fe(III) salt and SST-Fe(II) salt under anaerobic conditions (entries 1 and 2), MUFA trans isomer was detected at <1% with Fe(II) salt under anaerobic condition. With the reduced form of the peptide and Fe(II) salt still the reactivity for isomerization was low (entry 3). Instead, red-SST with Fe(III) salt under anaerobic conditions gave the most effective isomerization reaction, reaching 30% after 24 h incubation (entry 4). In aerobic conditions the same experiment gave isomerization, although at low extents (5%) (entry 5). 

When soybean lecithin liposomes are used, the reactivity of polyunsaturated fatty acids (PUFA) was followed-up, and the scenario changed in particular evidencing the consumption of PUFA due to peroxidation processes under aerobic conditions, as depicted first for bleomycin-Fe complexes [20]. The role of iron salt is important for the reactivity of the system, since SST under aerobic conditions but without Fe salts induced a slight peroxidation process and no isomerization after 24 h (entry 6, Table 1). The reactivity of PUFA-containing vesicles in the presence of both SST and Fe(II) salt increased toward the peroxidation of polyunsaturated residues, shortening the reaction time to 8 h instead of 24 h to detect an almost total PUFA consumption (96%, see entry 7, Table 1). At this time, the strong PUFA consumption did not allow for evidencing any isomerization process. Lowering Fe(II) concentration to 30 μM the peroxidation process decreased to ca. 75% (entry 8). When the low concentration of Fe(II) salt is used with red-SST, peroxidation slightly increased compared to the cyclic form of the peptide (entry 9). Finally, with 30 μM Fe(III) salt the peroxidation process occurred both with SST and red-SST, however the latter was found to be more efficient (cfr., entry 10 and entry 11). 

Further work is needed for the full understanding of the complex reactivity involving metal-sulfur adducts in liposomes, taking into account that the different isomerization yields can be also attributed to the diffusion of reactive radical species within the lipid bilayer. It is worth mentioning that the interaction of SST with its membrane receptors [2,5,6] brings this peptide in close contact with the lipid bilayer, therefore its reactivity with lipids cannot be ruled out. The present results obtained with the biomimetic model of liposomes using SST or its reduced form highlight the role of sulfur-containing biomolecules as trigger of oxidative radical-based processes which can transform unsaturated fatty acids, which are important constituents of cell membranes. This is a very interesting reactivity, which should be taken into account since lipid-peptide bioconjugation is used in liposome technology [29]. Lipid peroxidation and isomerization have been already evidenced for antitumoral drugs [20], and nowadays the oxidative aspect is considered not only as a side effect induced by anticancer therapy, but also as a condition to create oxidant-antioxidant unbalance in cancer cells [30]. This is connected with the metal redox state of the cellular environment that can be an important trigger of radical reactions, as shown for the reactivity of copper complexes [31,32]. Thiol compounds are reactive species in this context as in the new model here proposed for SST, expanding the actual knowledge of the molecular properties of this peptide for pharmacological applications. 

## 3. Conclusions

In this article we described the entrapment of SST in a lipid formulation that increased of 10-folds the stability of the peptide in human plasma. These results are promising for application as a drug delivery system by extension to appropriate experiments in biological context, using cells or murine models, which are ongoing. Furthermore, the inclusion of SST or its acyclic derivative in liposome vesicles has been used as biomimetic model in the presence of iron salts, for evidencing the reactivity between the peptide and its lipid carrier with thiyl radical formation and induction of lipid isomerization/peroxidation processes. New molecular insights are provided that can be developed further for synergic pharmacological strategies.

## 4. Materials and Methods 

Somatostatin (SST) and its acyclic form were received from Bachem, Germany and used with no further purification. POPC and l-α-phosphatidylcholine from soybean were obtained from Avanti Polar Lipids, USA and used without further purification. Formic acid and ammonium formate were purchased from Sigma-Aldrich, Milan and used as received. Acetonitrile, sodium citrate, citric acid and absolute ethanol were bought from Sigma-Aldrich, Milan and used without further purification. 

### 4.1. Preparation of Nanoemulsions (NE)

To prepare 10 mL of NE, 0.2 g of l-α-phosphatidylcholine (2% *w*/*v*) were dissolved in absolute ethanol, placed in a round-bottom flask and then the solvent was removed under reduced pressure to yield a thin and uniform lipid film on the wall of the flask. Five milligrams of SST (0.5 mg/mL) and 72.8 mg of sorbitol (2 mM) were dissolved in 10 mL of 0.1 M citrate buffer. The SST solution was then added to the lipid film and the flask content was maintained under agitation with vortex for 5 min. The so-obtained NE was freeze-dried and, when needed for further experiments, reconstituted with 10 mL of mq water.

### 4.2. LC/MS Analysis

Agilent 1260 Infinity automated LC/MS purification system was used, and the analyses were run with a reverse phase C-18 column (ZORBAX SB-C18 Rapid Resolution HT 2.1 × 50 mm 1.8 micron 600 Bar). A Phenomenex HPLC guard cartridge was used as well. The mobile phases were (A) H_2_O + 0.1% formic acid and (B) Acetonitrile + 0.1% formic acid. The samples were eluted with a linear gradient of B from 5% to 90% in 15 min, B was then decreased to 5% in 5 min (20 min) and kept so for 5 min (25 min).

### 4.3. In Vitro Release Experiments in Buffer

The release experiment was designed to analyze not only the directly released SST (detected in the medium) but also the amount of non-released SST, still entrapped in the lipid droplets (indirect analysis). Two different protocols were followed: (1) the freeze-dried NE was reconstituted in citrate buffer pH 5.0 0.1 M to start the release experiment and after each time point the analyses of the SST present in the resulting aqueous and lipid phases were carried out as described below; (2) after reconstitution in water, the NE was immediately centrifuged, the lipid fraction was isolated and resuspended in fresh citrate buffer pH 5.0 0.1 M to start the experiment. SST-containing NE were kept in a horizontal shaker at 37 °C (stirring rate 100 rpm). Reached the time point (0 min, 1 h, 3 h, 6 h, 24 h), three samples for each protocol of 0.2 mL were collected, centrifuged (15,000 rpm × 5 min × 4 °C) and 50 µL of supernatant were directly analyzed via LC/MS (direct analysis). The remaining lipid pellets were then dissolved in a mixture of hexane/formate buffer pH 3.0 (0.2 mL each, total volume 0.4 mL), vortexed for 5 min and after 10 min 50 µL of aqueous phase were withdrawn and analyzed with LC/MS (indirect analysis). The experiment was carried out three times for statistical analysis.

### 4.4. In Vitro Release Profile in Human Plasma

Two different protocols were followed according also to literature [33]: (1) the freeze-dried NE was reconstituted directly in human plasma to start the release experiment; (2) after reconstitution in tridistilled water, the NE was immediately centrifuged, the lipid fraction was isolated and suspended in human plasma to start the experiment. SST-containing NE were kept in a horizontal shaker at 37 °C (stirring rate 100 rpm). Reached the time point (0 min, 1 h, 3 h, 6 h, 24 h), three samples of 0.2 mL for each protocol were collected and centrifuged (15,000 rpm × 5 min × 4 °C). To 100 µL of the so-obtained supernatant were added 100 µL of TCA 6% (final concentration of TCA 3%) and the product was placed in ice bath for 10 min and centrifuged again (15,000 rpm × 5 min × 4 °C). 100 µL of the resulting supernatant were collected, quenched with a solution of NaOH 1 M and analyzed via LC/MS [1]. The indirect analysis was not performed due to incompatibility with the HPLC column.

### 4.5. Liposome Experiments

Large unilamellar vesicles were prepared with known methodologies [26]. Briefly, POPC or soybean lecithin were dissolved in chloroform and then evaporated to a thin film in a test tube under argon stream. In the next step the tube remained under vacuum for 30 min. Degassed water was added and multilamellar vesicles were formed by vortexing under argon atmosphere for 7 min. In order to obtain the LUVET vesicles of 200 nm diameter the emulsions were extruded by passage for 19 times through two polycarbonate membranes with the specific pore dimension. In a 4 mL vial 2 mM vesicle suspension was added together with the iron complex (0.1 mM), and the vial was kept under incubation at 37 °C adding somatostatin solution (0.1 mM) dropwise by a syringe pump (0.5 mm/min). The reaction mixture was worked up after the reported time (8 or 24 h) by adding 2:1 chloroform/methanol (1 mL) to extract lipids. The organic phase was dried over anhydrous sodium sulfate and evaporated under vacuum at room temperature to dryness. The resulting phospholipid extract was then treated with 0.5 M KOH/MeOH for 10 min at ambient temperature, converting them to the corresponding fatty acid methyl esters (FAME). The reaction was quenched with the addition of tridistilled water and an extraction with n-hexane followed. The organic layer containing the corresponding FAME was analyzed by GC under known conditions to examine cis and trans fatty acid isomers [25,27]. For the experiments under anaerobic conditions, all the solutions were degassed with argon for 15 min and the addition of all reagents took place under an argon stream. Anaerobic conditions were maintained during the incubation period by creating pressure of argon inside the reaction vial.

## Figures and Tables

**Table 1:** Formation of trans isomers of monounsaturated fatty acid (MUFA) and consumption of polyunsaturated fatty acids (PUFA) residues^1^ in liposome aqueous suspensions (2 mM) treated with 100 μM SST/red-SST and 100 μM Fe salts at 37 °C (n = 3 of each experiment).

Entry	Liposome	O_2_	Peptide	Fe(II)	Fe(III)	*trans*-18:1 (%)	*trans*-18:2 (%)	*PUFA Consumption (%)*
1	POPC ^2^	no	SST		x	tr		
2	POPC ^2^	no	SST	x		0.9 ± 0.1		
3	POPC ^2^	no	red-SST	x		0.7 ± 0.2		
4	POPC ^2^	no	red-SST		x	29.9 ± 0.2		
5	POPC ^2^	yes	red-SST		x	5 ± 0.3		
6	soybean lecithin ^3^	yes	SST			nd	nd	2.2 ± 1.4
7	soybean lecithin ^3^	yes	SST	x		tr	tr	95.9 ± 0.3
8	soybean lecithin ^3^	yes	SST	x^4^		0.3 ± 0.0	0.2 ± 0.1	75.5 ± 0.4
9	soybean lecithin ^3^	yes	red-SST	x^4^		0.4 ± 0.1	0.5 ± 0.1	84.1 ± 2.6
10	soybean lecithin ^3^	yes	SST		x^4^	tr	tr	35.9± 0.2
11	soybean lecithin ^3^	yes	red-SST		x^4^	tr	tr	92.3± 0.4

Nd = not detectable; tr = traces (<0.1%) ^1^ The fatty acid residues were obtained from the liposome suspension after lipid extraction and transesterification, as described in the Materials and Methods. ^2^ 24 h incubation. ^3^ 8 h incubation. ^4^ incubation with 30 μM Fe(II) salt.
